# DAMPs released by pyroptotic cells as major contributors and therapeutic targets for CAR-T-related toxicities

**DOI:** 10.1038/s41419-021-03428-x

**Published:** 2021-01-27

**Authors:** Tian Deng, Chao Tang, Guizhong Zhang, Xiaochun Wan

**Affiliations:** 1 Center for Protein and Cell-Based Drugs, Institute of Biomedicine and Biotechnology, Shenzhen Institutes of Advanced Technology, Chinese Academy of Sciences, 518055 Shenzhen, People’s Republic of China; 2 Guangdong Immune Cell Therapy Engineering And Technology Research Center (No. 2580 [2018]), Shezhen, People’s Republic of China; 3 Shenzhen BinDeBioTech Co., Ltd., Floor 5, Building 6, Tongfuyu Industrial City, Xili, Nanshan, 518055 Shenzhen, People’s Republic of China

**Keywords:** Cell death and immune response, Cancer immunotherapy, Inflammatory diseases

## Abstract

CAR-T transfer, recently well-developed immunotherapy, has offered substantial benefit to more and more patients with advanced cancers. However, along with growing experience in the clinical application comes the increasing awareness of the potentially fatal adverse effects, most notably cytokine release syndrome (CRS) and neurotoxicity. Understanding the mechanisms underlying these toxicities can help to improve therapeutic outcomes. Recent findings highlight the importance of monocyte/macrophage in CAR-T-related toxicities (CARTOX) and shed light on a novel mechanism mediated by damage-associated molecular patterns (DAMPs) released from pyroptotic cells. Therefore, this review summarizes these findings and provides practical guidance to the management of CARTOX.

## Introduction

Recently, chimeric antigen receptor T-cell (CAR-T) therapy has shown promising efficacy in refractory B-cell malignancies and brought hope for the treatment of other advanced cancers^1,2^. CAR-T therapy induces a rapid immune response and lasts for months or years, but also leads to certain toxicities like cytokine release syndrome (CRS) and neurotoxicity, which can be severe or even fatal^3,4^. So it is exigent to understand the mechanisms of these side effects and to develop strategies to reduce or eliminate therapy-induced toxicities.

CRS is a potentially life-threatening toxicity that can be triggered by infections (influenza^5^, COVID-19 ^6^), certain drugs, and immunotherapy, especially those involve T cells^7^. CRS is non-antigen-specific toxicity caused by high levels of immune activation^8^. It is associated with elevated circulating levels of several core cytokines including interleukin (IL)-6 ^8^. Hence, immunosuppression using tocilizumab, an anti-IL-6 receptor (IL-6R) antibody, with or without corticosteroids, can mitigate CRS^3,8^. However, since early and aggressive immunosuppression could limit the efficacy of the immunotherapy^8,9^, approaches that can address life-threatening complications of CRS without compromising CAR-T efficacy are urgently needed.

Unlike CRS, which is well understood, the pathophysiology and treatment of neurotoxicity have remained elusive. Although the association between IL-6 and the development of neurotoxicity has been investigated in several clinical experiences^10–12^, targeting IL-6R has not been shown to be effective for neurotoxicity treatment^4,13^. Therefore, more studies are required to evolve our understanding of the mechanisms underlying such CAR-T-related toxicities (CARTOX), and to identify more predictive biomarkers of severity and attractive therapeutic targets. Recent findings highlight the importance of monocyte/macrophage in CARTOX^14,15^, and shed light on a novel mechanism mediated by damage-associated molecular patterns (DAMPs) released from pyroptotic cells^16^. Therefore, this review summarizes these findings and provides practical guidance to the management of CARTOX.

### Monocytes/macrophages: the key mediator in CARTOX

Although the clinical manifestations of CARTOX are easily recognized, the detailed mechanisms still remain unclear. The previous study had demonstrated that high levels of cytokines contribute to both CRS and neurotoxicity by activating endothelial cells^11,17^, yet the source and precise function of these cytokines are ill-defined. Recently, two groups reported cytokines released from myeloid but not from CAR-T cells are the main cause of CRS and neurotoxicity^14,15^. One of the researches used SCID-beige mice with a high tumor burden, so as to initiate CRS within a few days. They found CAR-T cells activated by the tumor cells could recruit and activate macrophages through CD40L–CD40 interaction. Activated macrophage releases a large number of inflammatory mediators that have been described in CRS on clinical studies, including IL-6 and IL-1β, which exacerbate CRS. Modulation of macrophage function or IL-1 signaling blockade abrogates CRS-related mortality, signifying the importance of macrophage in CRS and suggesting IL-1 as a new potential target to alleviate CRS severity^15^. Using humanized triple transgenic NSG mice that can more completely recapitulate the CARTOX seen in humans, Norelli et al. confirmed IL-1 and IL-6 are produced by monocytes and serve as key contributors in CARTOX. Monocyte depletion or IL-1R blockade using anakinra protects mice from both lethal CRS and neurotoxicity, however, pre-emptive use of IL-6R antagonists can only prevent CRS but not neurotoxicity^14^, suggesting different priorities in the contribution for CARTOX between IL-1 and IL-6. IL-1 is a gatekeeper cytokine critically involved in many events related to inflammation^18^. IL-1 release precedes IL-6 by 24 h^14^, thereby is reasonable to make a more critical contribution to CARTOX, especially neurotoxicity. Collectively, these findings update our understanding of the sources of inflammatory cytokines and mechanisms for CARTOX and highlight macrophage as the key contributor for both CRS and neurotoxicity. Therefore, the activation and regulation of macrophages in the tumor microenvironment during CAR-T therapy should be the focus of future studies to find new targets for alleviating side effects and making CAR-T therapy safer.

### DAMPs: endogenous triggers for macrophage activation

Macrophages are an important group of innate immune system, existing in almost all tissues. They are differentiated from circulating monocytes^19^ and have important roles in the control of inflammation and infection^20^. Pathogen-associated molecular patterns (PAMPs) and damage-associated molecular patterns (DAMPs) are two major groups of macrophage triggers, which are released from invading pathogens and damaged or dying cells, respectively.

DAMPs also referred to as “danger”-associated molecular patterns, are endogenous immunogenic molecules released upon “danger” situations such as tissue damage or cellular stress. There are basically two categories of DAMPs according to the location, from the extracellular matrix (Decorin, Heparan sulfate, Fibrinogen, etc.) or intracellular compartments (HMGB1, ATP, HSP, etc.)^21,22^. DAMPs are recognized mainly by PRRs and trigger macrophage activation^22^, thereby be crucially involved in many inflammatory diseases^21,22^.

As recently reported, macrophages are involved in CARTOX development^14,15^, but the detailed mechanisms for macrophage activation remain to be determined. During CAR-T therapy, large amounts of cell death might cause DAMPs leakage and thereby trigger macrophage activation. However, this was not proven until this year.

### Pyroptosis and subsequent DAMPs leakage trigger macrophage activation resulting in CARTOX

Pyroptosis is a form of inflammatory programmed cell death, characterized by cell swelling, lysis, and the release of many inflammatory factors as well as DAMPs^23^. Dying cells activate pyroptosis through the following three main approaches: (i) GSDMD (gasdermin D)-dependent activation mediated by caspase 1/4/5/11 ^24^; (ii) GSDME-dependent activation mediated by caspase 3 ^25,26^ and (iii) GSDMB-dependent activation mediated by lymphocyte-derived granzyme A^27^. Activated gasdermins release the novel segment with membrane pore-forming activity and leading to pyroptosis^23,28^.

**Fig. 1:**
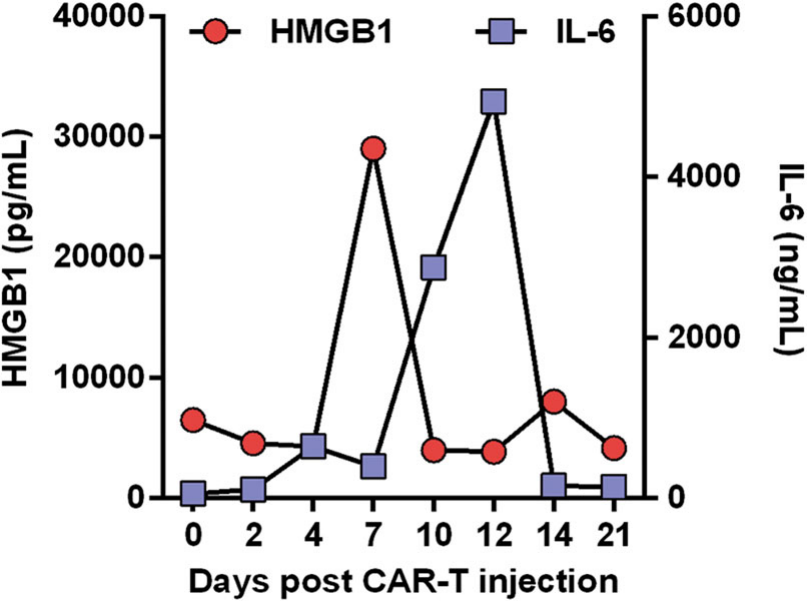
Time course of HMGB1 and IL-6 release post-CAR-T injection. Sera HMGB1 and IL-6 levels of 10 acute lymphoblastic leukemia patients with CAR-T therapy were detected using ELISA kit (SEA399Hu, Cloud-Clone Corp.) and CBA kit (551811, BD) respectively. The representative time-course is presented.

A recent study reported CAR-T cells can induce GSDME-mediated target cell pyroptosis, which resulted in CRS^16^. They found CAR-T cells release granzyme B into tumor cells to activate caspase 3, causing the subsequent activation of GSDME and pyroptosis. Pyroptotic tumor cells release large amounts of DAMPs, HMGB1 and ATP in particular, which activate macrophages and induce the release of IL-1β and IL-6, causing CRS. Correspondingly, a higher level of GSDME in primary B-ALL leukemia cells was associated with a more severe case of CRS in patients who accepted CD19-CAR-T treatment^16^, signifying the importance of pyroptosis in CRS. Although it has not been determined yet, however, the dramatic induction of IL-1β strongly indicates the involvement of pyroptosis–DAMPs axes in neurotoxicity. Taken together, this study reveals a possible mechanism that how CAR-T therapy and tumor cells themselves trigger the macrophage-mediated toxicities and highlights the key involvement of pyroptosis–DAMPs axis in CARTOX. In line with these findings, our data further confirm this view and suggest HMGB1 serves as a predictive biomarker and attractive therapeutic target for CRS since 7/10 patients who received CAR-T cells induce high levels of HMGB1, which preceded IL-6 release (Fig. 1).

**Fig. 2:**
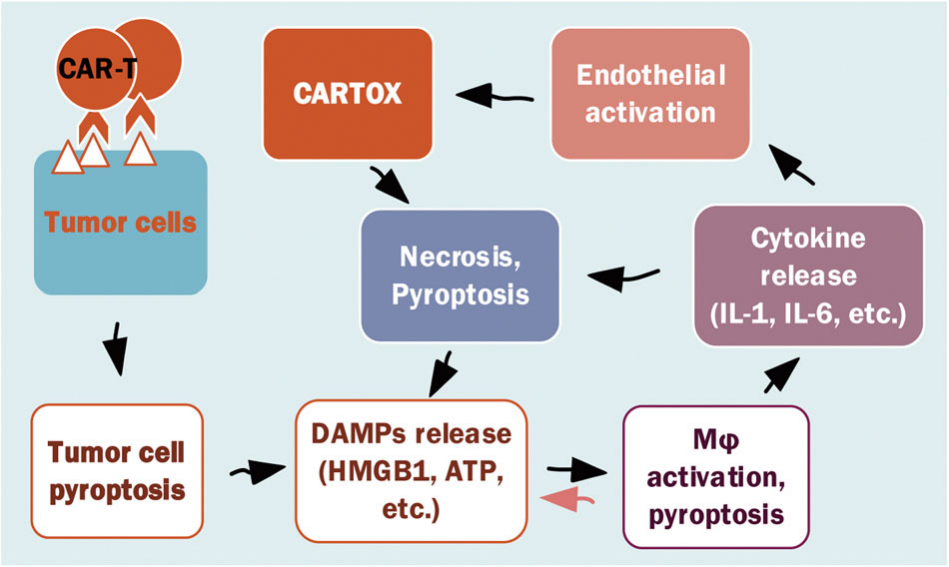
Proposed pathomechanism of CARTOX. CAR-T cells recognize and trigger tumor cells in pyroptosis. Pyroptotic cells release large amounts of DAMPs, which activate macrophages (Mφ) with subsequent cytokine release, resulting in endothelial activation and CARTOX. High levels of cytokines impair other cell functions and even cause necrosis or pyroptosis, further increase DAMPs leakage, which forms a vicious circle that leads to more severe CARTOX. In addition, DAMPs, such as HMGB1, could be endocytosed and induce macrophage pyroptosis, further increasing DAMPs release and exacerbating CARTOX.

HMGB1 has recently attracted much attention for its pro-inflammatory activity and potential clinical applications in many inflammatory diseases^29^. It is a nonhistone chromatin-binding protein and participates in many important nuclear processes in a steady-state, such as replication, DNA repair, and transcription^30^. In the context of tissue or cell stress, HMGB1 is mobilized into the cell cytoplasm or released to the extracellular space to drive the inflammatory responses as a DAMP^30,31^. HMGB1 is recognized by several receptors, including TLR2, TLR4, TLR9, and RAGE^32–34^. Recent studies revealed HMGB1 can induce macrophage activation through binding to TLR4 ^35^, and trigger macrophage pyroptosis through RAGE/dynamin-dependent endocytosis^36^. Pyroptotic macrophages could release more DAMPs to favor further immune activation and cytokine release, which might form a vicious circle leading to more severe CARTOX. Additionally, high levels of cytokines might induce necrosis or pyroptosis in tissue cells^37,38^, resulting in severe DAMPs leakage which can further exacerbate CARTOX. That’s to say, CARTOX is a set of macrophage-dependent complications, in which DAMPs released by pyroptotic cells as upstream trigger have critical roles. During CARTOX, several processes including pyroptosis, DAMPs release, macrophage activation, and cytokine release form a loop that drives CARTOX more severely (Fig. 2). Strategies capable of breaking down links in this loop might be exploited to manage CARTOX, as IL-6R or IL-1R blockade can mitigate CRS or neurotoxicity. However, both IL-6 and IL-1 are only two identified macrophage-derived cytokines that lie downstream of inflammatory events that result in CARTOX, their inhibition may be insufficient hence ineffective. Therefore, targeting pyroptosis to reduce DAMPs release, or directly targeting DAMPs, the upstream specific triggers for macrophage activation during CAR-T therapy, might be a more prudent approach for CARTOX treatment.

## Conclusion

CARTOX remains a common challenge of CAR-T therapies. Recently, our understanding of the molecular mechanisms governing CARTOX has evolved substantially. The identification of pyroptosis–DAMPs–macrophage loop involvement opens up new avenues by which CARTOX can be better predicted and treated. It is reasonable to speculate that blocking the executors of pyroptosis (such as GSDME), consuming the DAMPs released from pyroptotic cells, or improving CAR-T design to cause tumor cell apoptosis but not pyroptosis may be possible strategies to reduce CARTOX.
